# Development and validation of FinTox: A new screening tool to assess cancer‐related financial toxicity

**DOI:** 10.1002/cam4.7306

**Published:** 2024-08-07

**Authors:** Hoda Badr, Jessie Han, Martha Mims

**Affiliations:** ^1^ Department of Medicine Baylor College of Medicine Houston Texas USA

**Keywords:** financial hardship, financial toxicity, measure, psychometric validation, screening tool

## Abstract

**Purpose:**

This study aimed to develop and validate FinTox, a concise tool for screening and managing financial toxicity in oncology settings.

**Methods:**

Development involved qualitative interviews with healthcare providers and patients, and feedback from a 7‐member expert panel resulting in a 5‐item measure that evaluates financial strain, psychological responses, and care modifications. Psychometric evaluations examined factor structure, internal consistency, test–retest reliability, and concurrent and convergent validity. Associations between FinTox scores and sociodemographic/medical factors were also analyzed using univariate and multivariable regression models.

**Results:**

Twelve healthcare providers and 20 patients were interviewed, and 268 patients (69.8% female, 47.4% non‐Hispanic White) completed surveys including FinTox, the Comprehensive Score for Financial Toxicity (COST), health‐related quality of life (HRQOL) measures, and sociodemographic questions. FinTox demonstrated excellent internal consistency (Cronbach's alpha = 0.90) and test–retest reliability (ICC = 0.95). Significant correlations with the COST (*r* = −0.62, *p* < 0.001) and HRQOL measures corroborated content and convergent validity. Diagnostic accuracy was evidenced by a sensitivity of 72.3%, specificity of 85.2%, positive predictive value of 83.2%, and negative predictive value of 70.3%. Higher FinTox scores were also associated with receiving care at a safety‐net hospital, Black race, household income <600% of the federal poverty level, and Stage 4 cancer.

**Conclusion:**

FinTox's robust psychometric properties and diagnostic accuracy position it as a reliable tool for detecting financial toxicity. Future research should evaluate its responsiveness to changes over time and integration into clinical workflows.

## Graphical abstract

This study developed and validated FinTox, a pragmatic 5‐item tool for efficiently screening and triaging cancer patients experiencing financial toxicity in oncology clinical care settings.

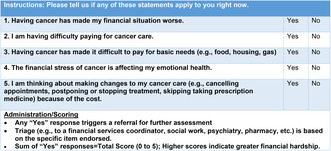

## 1 INTRODUCTION

The trajectory of cancer survivorship in the United States is on a significant upward trend, with forecasts of 22.5 million survivors by 2032.^1^, ^2^ This increase, fueled by an aging population and advances in cancer detection and treatment, coincides with escalating treatment costs. Cancer is one of the most expensive medical conditions to treat,^3^ with costs expected to exceed $246 billion by 2030, owing to the growing use of sophisticated treatments like chemotherapy, biologics, and targeted therapies.^4^ Cancer patients increasingly face higher out‐of‐pocket expenses due to rising healthcare insurance deductibles, copayments, and coinsurance^5^, ^6^, ^7^, ^8^, ^9^; and, financial impacts vary across healthcare systems. Public safety‐net systems aim to minimize direct medical costs, yet resource constraints can delay treatment and limit access to advanced therapies. Patients in these settings might also incur additional financial burdens, such as transportation costs, exacerbating their economic hardship. Conversely, private practice systems often offer broader and more immediate treatment options but can lead to significant financial difficulties due to high treatment fees and inconsistent insurance coverage. Thus, there is a crucial need for standardized screening tools to evaluate the financial challenges patients encounter across healthcare settings, facilitating early triage and referral to appropriate financial services and support.

In this challenging and evolving landscape, “financial toxicity” has emerged as a key concept. The term encapsulates the negative financial consequences of cancer and its treatment, encompassing escalating out‐of‐pocket costs, income reduction due to work absences or job loss, mounting debts, and challenges settling medical bills.^10^ It has also been used to describe material financial hardship (e.g., bankruptcy) and subjective financial distress.^11^, ^12^ Many patients experience financial toxicity while navigating the cancer care system. Recent research reveals a stark reality: 25.3% of cancer survivors experience material financial hardship, 34.3% suffer from subjective financial distress, and 26% endure behavioral financial hardship such as delaying or forgoing medical care due to cost.^13^, ^14^ Significant disparities have also been observed, with racial and ethnic minorities, those of younger age or in lower socioeconomic groups, and patients with advanced‐stage cancer disproportionately affected by financial toxicity.^15^, ^16^ Financial toxicity has also been shown to adversely affect patient decision‐making, adherence to treatment, and HRQOL.^17^, ^18^ Oncologists and other healthcare providers, who are in regular contact with patients, insurance providers, and cancer center administrators, play a pivotal role in addressing this issue. Their unique position enables them to facilitate discussions and actions among these stakeholders, contributing to the mitigation of financial toxicity. However, challenges such as time limitations, lack of knowledge about hospital and community financial assistance resources, and discomfort in discussing financial matters often impede effective communication between patients and oncologists, hindering the identification and management of financial toxicity.^11^ Overcoming these barriers is essential to ensure appropriate identification and management of financial toxicity.

Screening is a pivotal initial step in connecting patients experiencing or at risk of developing financial toxicity with pertinent clinical and community resources. Implementing universal screening helps mitigate bias and reduce patient stigma. However, existing patient‐reported outcome measures (PROMs) for financial toxicity, such as the 11‐item Comprehensive Score for Financial Toxicity (COST)^10^ often fall short in clinical utility due to their length and complexity, rendering them impractical for fast‐paced clinical environments where quick and straightforward tools are paramount. Moreover, the COST was initially validated in a cohort mainly comprising insured, middle‐aged patients with solid tumors. Consequently, it may not fully capture the experiences of uninsured patients or those encountering unique financial challenges. Other cancer‐specific financial measures like the Functional Assessment of Cancer Therapy‐General (FACT‐G) Financial Well‐Being Subscale, the European Organization for Research and Treatment of Cancer (EORTC) QLQ‐C30 financial impact subscale, the Breast Cancer Finances Survey Inventory,^19^ the Socioeconomic Well‐being Scale (SWBS),^20^ and the InCharge Financial Distress/Financial Well‐being Scale,^21^ exhibit substantial variation in development, validation, and ease of implementation in clinical practice. This highlights a critical gap in current practices and underscores the pressing need for a brief, easily administered, and actionable screening tool for use in cancer clinical care settings.

Addressing this need, this study introduces the Financial Toxicity (FINTOX) screening measure, which was developed expressly for assessing financial toxicity in oncology clinical settings. FINTOX is designed as a concise, easily administered tool to enable efficient identification of patients experiencing financial toxicity. It offers a practical solution for busy clinical environments where quick, straightforward tools are essential for effective patient care. The measure seeks to facilitate identification of patients in need of financial assistance and referral to financial navigation and counseling resources, with the overall goal of improving patient‐centered care.

## 2 METHODS

### 2.1 Study sample and procedures

The study was approved by Baylor College of Medicine's Institutional Review Board and written informed consent was obtained. Eligibility criteria included: cancer patients undergoing treatment or survivors within 3 years of completing standard definitive therapy, age 18 or older, English or Spanish fluency, and capability to consent. Healthcare workers were also eligible. Recruitment occurred at two hospital affiliates of the Dan L Duncan Comprehensive Cancer Center (DLDCCC)—Baylor St Luke's Medical Center (BSLMC; private practice) and Harris Health Smith Clinic (HHSC; safety‐net).

This study adhered to methodologies outlined by the International Society for Pharmacoeconomics and Outcome Research (ISPOR) Patient Reported Outcomes Content Validity Good Research Practices Task Force,^22^, ^23^ and the Consensus‐based Standards for the selection of health Measurement Instruments (COSMIN) study.^24^ It encompassed two phases: development and validation.

Phase 1, Development, comprised concept elicitation and coding, followed by item generation. Concept elicitation and coding entailed semi‐structured interviews conducted by research staff with healthcare workers and cancer patients. Healthcare workers received invitations via email or in person, while patient recruitment occurred during clinic visits. Conducted in English and Spanish, these interviews lasted approximately 60 min, with participants receiving a $50 gift card. The interview guide for healthcare providers delved into perceptions of patient financial navigation needs, resource adequacy, the feasibility and practicality of universal financial screening, and implementation preferences (see Appendix S1). The patient‐focused guide explored the impact of cancer‐related costs on financial and psychological well‐being, healthcare decisions, daily life, and coping strategies, along with information and support needs (see Appendix S2). All interviews were audio‐recorded, transcribed, and coded.

Item generation entailed the research team developing a survey item bank from themes identified in the qualitative interviews and translating the items into Spanish. Two rounds of cognitive interviewing with the 20 patients who participated in the interviews were conducted to refine the items' face and content validity. The first round tested participants' understanding of the survey items, focusing on clarity, relevance, and language simplicity. Feedback led to modifications to eliminate ambiguities and enhance item relevance. The second round retested these revised items to ensure the changes effectively addressed initial concerns without introducing new issues. The team then collaborated with a 7‐member expert panel (comprising four MDs, a clinical pharmacist, a social worker, and a financial navigator) to streamline the item bank, eliminating redundant or tangential items. The remaining items were ranked by clinical relevance, and the top five were selected for the FINTOX measure.

Phase 2, Validation, involved enrolling a new patient cohort to evaluate the internal and external validity of the FINTOX measure. Patients meeting the eligibility criteria were identified from electronic health records and invited to complete a cross‐sectional survey during routine clinic visits. This survey included sociodemographic questions, the FINTOX measure, and various HRQOL measures. Participants were compensated with a $10 gift card per completed survey. A subset of patients also completed test–retest follow‐up surveys, featuring only the FINTOX items, 7 days after the initial survey, receiving an additional $5 gift card for each completed follow‐up survey.

Measures.

### 2.2 Financial toxicity

The Comprehensive Score for Financial Toxicity (COST)^10^ is an 11‐item validated tool designed to assess financial toxicity, comprising two items on financial resources and eight on psychological responses. Scores range from 0 to 44, with lower scores indicating greater financial toxicity. Da Souza and colleagues established cut‐off values of ≥26 for no/mild, 14–25 for moderate, and 0–13 for severe financial toxicity.^25^ In this study, COST demonstrated a Cronbach's alpha of 0.91. We hypothesized a moderate to high negative correlation between the FINTOX and COST measures because lower COST scores indicate greater financial toxicity.

HRQOL was assessed using three measures to allow for a multifaceted evaluation of HRQOL in relation to financial toxicity. The 27‐item Functional Assessment of Cancer Therapy‐General (FACT‐G) evaluates physical, functional, emotional, and social well‐being, and was included to elucidate associations with general HRQOL domains. Scores range from 0 to 108, with higher scores indicating better quality of life.^26^, ^27^ The FACT‐G demonstrated high reliability in this study, evidenced by a Cronbach's alpha of 0.93. The second measure, the 14‐item Condensed Memorial Symptom Assessment Scale (CMSAS), assesses 11 physical and 3 psychological symptoms experienced in the past week, and was included to provide insights on the relationship between symptom burden and financial toxicity. Scores range from 0 to 4, with higher scores indicating more severe symptoms.^28^ The CMSAS showed a Cronbach's alpha of 0.89, indicating good internal consistency. The third tool, the 4‐item Perceived Stress Scale (PSS‐4), measures perceived unpredictability, uncontrollability, and overload in life.^29^ Scores range from 0 to 16, with higher scores denoting greater stress. The PSS‐4 was included because it captures the overall stress response (as opposed to specific symptoms) and the broader psychological impact of being in a financially toxic environment. In this study, the PSS‐4 had a Cronbach's alpha of 0.73, suggesting acceptable reliability. We hypothesized that FinTox scores would have a moderate negative correlation with FACT‐G scores and a moderate positive correlation with CMSAS and PSS‐4 scores.

Data on patients' sociodemographic and descriptive variables included age, gender, race/ethnicity, education, marital status, employment status, insurance type, household income, household size, and cancer type and stage. The federal poverty level (FPL) was calculated using annual household income and size, classifying individuals below 200% of the FPL as living in poverty.^30^, ^31^ Patients also reported their monthly out‐of‐pocket medical expenses. An adapted item from Meisenberg^32^ measured how frequently patients and doctors discussed cancer care costs on a 5‐point scale (1 = rarely to 5 = very frequently). A subsequent question asked patients to specify the main reason for not discussing costs, with options including perceived irrelevance to the doctor's role, time constraints, concerns about judgment, cost‐related therapy alterations, or doctor's perceived indifference to costs.

### 2.3 ANALYSIS

#### 2.3.1 Scale development phase

Participant interviews underwent rapid qualitative analysis.^33^ A structured coding template, derived from the interview guides, facilitated this process. Two trained analysts synthesized participant responses into case summaries, focusing on key domains of interest. These summaries were then organized into a matrix display, enabling systematic identification of response patterns, variations, and trends.^34^ To validate the findings, the process incorporated analyst triangulation and peer debriefing.^35^

#### 2.3.2 Scale validation phase

The study sample was characterized using descriptive statistics (Mean, Standard Deviation (SD), frequencies). FinTox's internal consistency was evaluated through a quantitative item analysis. Exploratory factor analysis (EFA) determined the measure's internal structure,^36^, ^37^ with Bartlett's test of Sphericity and the Kaiser–Meyer–Olkin's (KMO) measure assessing item correlation significance. Factors were identified using the Kaiser criterion (eigenvalues >1) and scree plot analysis, considering loadings >0.50 for statistical and practical relevance.^38^ Cronbach's alpha assessed internal consistency reliability. Redundancy was minimized by analyzing inter‐item correlations (IICs) and item‐total correlations (ITCs) to ensure items significantly contributed to the overall score. In addition, test–retest reliability was measured by calculating the Intraclass Correlation Coefficient (ICC) from initial and 7‐day follow‐up FINTOX scores, using a single‐rater, two‐way random effects model, with an ICC >0.7 indicating satisfactory consistency.^39^

Sensitivity and specificity of FinTox were evaluated by comparing it against COST's thresholds for moderate to severe financial toxicity. We categorized outcomes into four types: true positives (TP), where both FinTox and COST detected financial toxicity; false negatives (FN), where FinTox did not detect financial toxicity but COST did; true negatives (TN), where neither measure detected financial toxicity; and false positives (FP), where FinTox indicated financial toxicity whereas COST did not. Sensitivity, the proportion of actual positives correctly identified by FinTox, was calculated using the formula: TP/(TP + FN). Specificity, the proportion of actual negatives correctly identified, was calculated using the formula: TN/(TN + FP). Additionally, we calculated positive predictive value (PPV) and negative predictive value (NPV) to assess FinTox's clinical utility. PPV, the proportion of positive results that are true positives, was calculated as PPV = TP/(TP + FP). NPV, the proportion of negative results that are true negatives, was calculated as NPV = TN/(TN + FN).

To evaluate construct validity, associations between the FinTox and COST measures were examined. Convergent validity involved Pearson correlations between FinTox and HRQOL measures (FACT‐G, PSS‐4, CMSAS), expecting mild to moderate significant correlations. For known groups validity, univariable analyses (*t*‐tests, ANOVAs, Chi‐squares) identified sociodemographic groups differing in FinTox scores, considering variables like study site, age, gender, marital status, race, education, insurance type, employment status, income, and cancer stage. Significant variables were further examined using univariate regression, with FinTox score as the dependent variable. Predictors were independently assessed for inclusion in a stepwise multivariable linear regression model, using a *p* < 0.10 criterion for selection. Two‐tailed significance tests and 95% confidence intervals (CIs) were calculated at the *p* < 0.05 level.

#### 2.3.3 Sample size calculation

For the qualitative interviews, sample size was based on principles of thematic saturation, ceasing data collection when no new information emerges. Typically, a sample of 12 is considered adequate for this purpose.^40^ For the survey component, a sample of 268 was calculated to provide 80% power to detect a correlation coefficient of 0.24 between the FDST and the HRQOL instruments using a two‐sided significance level of 0.05. For the test–retest analysis, we calculated that a sample of 40 individuals provides 80% power to demonstrate excellent reliability (ICC of 0.80, assuming an ICC under the null hypothesis of 0.30) with a significance level of *p* < 0.05.

## 3 RESULTS

### 3.1 Scale development phase

Semi‐structured interviews involved 12 healthcare workers (6 oncologists, 3 nurses, a social worker, a financial navigator, and a clinical pharmacist) and 20 cancer patients. The patient cohort was predominantly female (70%), racially and ethnically diverse (60% African American, 10% Hispanic), and comprised diverse cancer types (breast–6, hematologic–4, gastrointestinal–4, lung–3, head and neck–2, kidney–1) and stages (I ‐ 4, II ‐ 5, III ‐ 7, IV ‐ 4). Average age was 62 years (SD = 7.93; range: 50–85 years).

Healthcare workers advocated for universal financial screening, emphasizing the need for brevity, focus on direct financial impacts of cancer, relevance to clinical care or referral options, and applicability across the cancer trajectory. Analysis of patient and provider interviews identified six financial hardship domains: material conditions (e.g., property sales, bankruptcy), financial strain/burden (e.g., paying for care, basic needs), psychological response (e.g., worry, distress), lifestyle changes (e.g., vacation cancelation, spending reduction), care plan modifications (e.g., reduced medication, skipped treatments), and financial support needs (e.g., information, assistance sources).

From the coded quotes within each domain, the research team created an initial bank of 31 items, categorized as follows: material conditions (5 items), financial strain/burden (6 items), psychological response (4 items), lifestyle alterations (4 items), care plan alterations (8 items), and financial support needs (4 items). Subsequently, cognitive interviews were conducted in two rounds with 12 of the 20 patients who participated in the qualitative interviews. In the first round, participants were asked to verbalize their thoughts while completing the survey, providing feedback on question meaning, relevance, readability, and cultural and linguistic suitability. Adjustments were made based on this feedback. The second round involved participants reviewing the revised survey, resulting in only minor further changes.

Drawing from healthcare worker interviews, the research team and a 7‐member expert panel, comprising 4 MDs, a clinical pharmacist, social worker, and financial navigator, refined the item bank. They focused on eliminating redundant or less relevant items and then ranked the remaining for clinical importance. The top five items, as shown in Table 1, include three on financial strain/burden, one on psychological response, and 1 on care plan changes. The panel unanimously recommended a simple yes/no response format, where each ‘yes’ equals 1 point. They felt that a binary format was best suited for rapid clinical assessments and straightforward interpretation by healthcare providers to facilitate referrals to support services such as financial counseling, social work, psychiatry, or pharmacy services based on the specific needs identified during the assessment. For research purposes, they also recommended the option of summing the ‘yes’ answers to calculate a total score, ranging from 0 to 5, with higher scores reflecting greater financial toxicity.

**TABLE 1:** FinTox screening measures: Items and scoring.

Instructions: Please tell us if any of these statements apply to you right now		
1. Having cancer has made my financial situation worse	Yes	No
2. I am having difficulty paying for cancer care	Yes	No
3. Having cancer has made it difficult to pay for basic needs (e.g., food, housing, gas)	Yes	No
4. The financial stress of cancer is affecting my emotional health	Yes	No
5. I am thinking about making changes to my cancer care (e.g., canceling appointments, postponing or stopping treatment, skipping taking prescription medicine) because of the cost	Yes	No
Administration/Scoring Any “Yes” response triggers a referral for further assessment Triage (e.g., to a financial services coordinator, social work, psychiatry, pharmacy) is based on the specific item endorsed Sum of “Yes” responses = Total score (0–5); higher scores indicate greater financial hardship		

### 3.2 Scale validation phase

#### 3.2.1 Sample descriptives

Two‐hundred sixty‐eight patients completed surveys, and a subset of 40 completed the test–retest survey. Table 2 indicates that most were female (69.8%), married (51.1%), and from racial/ethnic minority groups (45.1%). Over half (56.4%, *N* = 151) were unemployed or retired. A third (*N* = 90) had incomes below 200% of the FPL, and most (58.6%) had public healthcare coverage. The average monthly out‐of‐pocket medical expense was $365.57 (SD = $465.49; range: 0 to $3000). About 64.6% rarely or never discussed care costs with their oncologist, mainly believing it was not the oncologist's role (84.3%), fearing therapy changes due to cost (40.7%), and due to limited time during visits (39.3%). One‐hundred thirty (48.5%) patients had either moderate (*N* = 75) or high (*N* = 55) levels of financial toxicity based on the COST measure.

**TABLE 2:** Sample characteristics and mean FinTox scores across different sociodemographic factors.

Sociodemographic factors	*N* = 268	FinTox (Mean ± SD)	*p*‐value
Site			
BSLMC (private practice)	170 (63.4%)	0.71 ± 1.53	<0.001
HHSC (safety‐net)	98 (36.6%)	2.29 ± 1.87	
Age			
≤50 years	69 (25.7)	2.01 ± 2.10	<0.001
51–64 years	118 (44%)	1.42 ± 1.81	
65–74 years	54 (20.1%)	0.52 ± 1.23	
≥75 years	20 (7.5%)	0.4 ± 1.19	
Missing	7 (2.6%)		
Gender			
Female	187 (69.8%)	1.19 ± 1.81	0.190
Male	80 (29.9%)	1.51 ± 1.85	
Missing	1 (0.4%)		
Marital status			
Single	127 (47.4%)	1.69 ± 1.97	<0.001
Married	137 (51.1%)	0.92 ± 1.60	
Missing	4 (1.5%)		
Race/ethnicity			
White, non‐Hispanic	127 (47.4%)	0.62 ± 1.44	<0.001
White, Hispanic	58 (21.6%)	2.19 ± 2.05	
Black, non‐Hispanic	49 (18.3%)	2.06 ± 1.99	
Asian or Pacific islander	14 (5.2%)	0.93 ± 1.82	
Missing	20 (7.5%)		
Education level			
No college degree	141 (52.6%)	2.04 ± 2.03	<0.001
College degree	115 (42.9%)	0.69 ± 1.50	
Missing	12 (4.5%)		
Insurance type			
Private (through job or school)	89 (33.2%)	0.84 ± 1.62	<0.001
State/Federal health exchange (e.g., affordable care)	43 (16.0%)	2.07 ± 2.13	
Any public (e.g., Medicare, Medicaid, safety‐net hospital plan)	43 (16%)	1.44 ± 1.85	
Missing	22 (8.2%)		
Employment status			
Employed (full‐time or part‐time)	91 (34%)	1.21 ± 1.91	0.13
Unemployed	151 (56.3%)	1.50 ± 1.98	
Missing	26 (9.7%)		
% of federal poverty level (FPL)			
0%–199.99%	90 (33.6%)	2.69 ± 2.06	<0.001
200%–399.99%	43 (16.0%)	0.77 ± 1.66	
400%–599.99%	18 (6.7%)	0.94 ± 1.83	
600%–799.99%	15 (5.6%)	0 ± 0	
800%–999.99%	15 (5.6%)	0 ± 0	
≥1000%	13 (4.9%)	0 ± 0	
Missing	74 (27.6%)		
Cancer stage			
Stage I	50 (18.7%)	0.86 ± 1.71	0.01
Stage II	43 (16.0%)	0.93 ± 1.73	
Stage III	35 (13.1%)	1.60 ± 1.97	
Stage IV	62 (23.1%)	2.00 ± 2.16	
Missing	78 (29.1%)		

Abbreviations: BSLMC, Baylor St Luke's Medical Center; HHSC, Harris Health Smith Clinic.

#### 3.2.2 Scale descriptives

**FIGURE 1:**
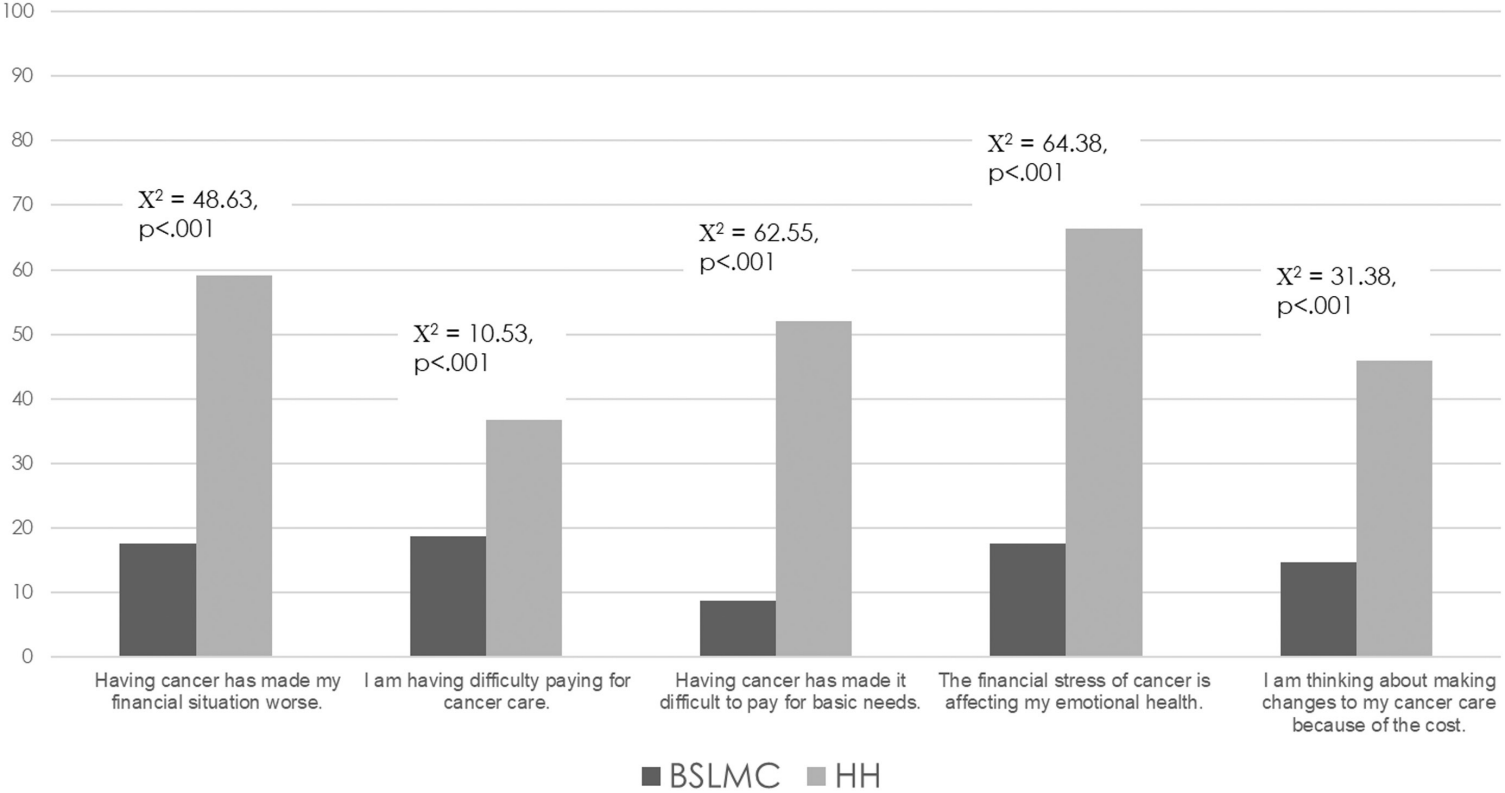
Chi‐square analysis showing percent of patients endorsing FinTox items at each study Site.

The average FinTox score was 1.44 (SD = 1.92, range 0 to 5); 113 of 268 patients (42.2%) endorsed at least one item, and 33 (12.3%) endorsed all five. The most commonly endorsed items were “The financial stress of cancer is affecting my emotional health” (35.4%) and “Having cancer has made my financial situation worse” (32.8%). Endorsement rates for the remaining three items varied between 24.6% and 26.1%. Table 2 shows mean FinTox scores across different sociodemographic and medical characteristics. Chi‐square analyses assessed the percentage of patients endorsing each FinTox item by study site, with Figure 1 indicating higher endorsement rates for all items among patients at HHSC.

#### 3.2.3 EFA results

The results of Bartlett's test of sphericity indicated that the correlation matrix was not random, *χ* ^*2*^(190) = 837.40, *p* < 0.001, and the Kaiser Meyer Olkin (KMO) statistic was 0.89. One factor was clearly identified upon inspection of the scree plot, with an eigenvalue of 3.62, explaining 72.37% of the variance. All 5 FinTox items had factor loadings >0.60, with communalities between 0.79 and 0.90.

#### 3.2.4 Reliability analysis

Cronbach's alpha for the 5‐item measure was 0.90 (95% CI = 0.89–0.92), demonstrating excellent internal consistency. The mean IIC of the 5‐item FinTox measure was 0.65 (Range = 0.55–0.79), and the mean ITC was 0.76 (Range = 0.69–0.83), demonstrating nonredundancy and good construct validity. Test–retest analysis revealed an ICC of 0.95 (95% CI, 0.90–0.97).

#### 3.2.5 Sensitivity and specificity

Sensitivity analysis revealed a rate of 72.3%, with 94 TP and 46 FN. Specificity was found to be 85.2%, calculated from 109 TN and 19 FP.

#### 3.2.6 NPV and PPV

The PPV was 83.2% (94 true positives out of 113 positive cases identified by FinTox). The NPV was 70.3% (109 TN out of 155 negative cases identified by FinTox).

#### 3.2.7 Construct and convergent validity

As Table 3 shows, as expected, FinTox was moderately negatively correlated with the COST (*r* = −0.62) and FACT‐G (*r* = −0.57) measures, and significantly moderately positively correlated with CMSAS (*r* = 0.50) and PSS‐4 (*r* = 0.49) scales (all *p*‐values < 0.01).

**TABLE 3:** Concurrent and convergent validity results.

Variable	1	2	3	4	5	Mean	SD
1 FINTOX	—	—	—	—	—	1.28	1.82
2 FACT‐G^a^	−0.57*	—	—	—	—	72.97	18.18
3 PSS‐4^b^	0.49*	−0.71*	—	—	—	6.26	3.17
4 CMSAS^c^	0.50*	−0.75*	0.53*	—	—	1.06	0.71
5 COST^d^	−0.62*	0.60*	−0.65*	−0.53*	—	24.00	11.62

* *p* < 0.01.

^a^ Functional Assessment of Cancer Therapy—General. Higher scores indicate greater quality of life.

^b^ 4‐item Perceived Stress Scale—Higher scores indicate greater perceived stress.

^c^ Condensed Memorial Symptom Assessment Scale—Higher scores indicate greater symptom burden.

^d^ Comprehensive Score for Financial Toxicity—Lower Scores indicate greater financial toxicity.

#### 3.2.8 Known groups validity

As Table 4 shows, univariable analyses revealed significant mean differences on FINTOX scores based on study site, age, marital status, race/ethnicity, education, type of insurance, household income, and cancer stage. As Table 4 also shows, univariate linear regression analyses revealed several sociodemographic factors that were associated with financial toxicity. However, in the final multivariable model, receiving care at a safety‐net hospital, Black race, a household income <600% of the FPL, and having Stage 4 cancer were identified as risk factors for greater financial toxicity. Being Asian, having a public health plan, and having a household income ≥600% of the FPL were protective factors.

**TABLE 4:** Univariate and multivariable linear regression analysis showing FinTox scores as a function of sociodemographic factors.

	Univariate			Multivariable		
Factor	Crude regression coefficients	95% CI	*p*‐value	Adjusted regression coefficients	95% CI	*p*‐value
Site						
BSLMC	Ref		<0.001	Ref		<0.001
HHSC	1.8	2.25 ± 1.40		2.11	1.34 ± 2.88	
Age						
≤50 years	Ref		<0.001			
51–64 years	−0.63	−1.18 ± −0.07				
65–74 years	−1.57	−2.23 ± −0.91				
≥75 years	−1.68	−2.62 ± −0.73				
Marital status						
Single	Ref		<0.001			
Married	−0.89	−1.35 ± −0.44				
Race/ethnicity						
White, non‐Hispanic	Ref		<0.001	Ref		<0.01
White, Hispanic	1.72	1.16 ± 2.25		0.04	−0.65 ± 0.74	
Black, non‐Hispanic	1.63	1.04 ± 2.22		1.11	0.42 ± 1.80	
Asian or Pacific islander	0.25	−0.74 ± 1.24		−1.27	−2.20 ± −0.33	
Education level						
No college	Ref		<0.001			
College degree	−1.35	−1.80 ± −0.90				
Insurance type						
Private (through job or school)	Ref		0.001	Ref		0.01
State/Federal Health Exchange (e.g., affordable Care)	1.23	0.56 ± 1.89		0.44	−0.31 ± 1.18	
Any public (e.g., Medicare, Medicaid, safety‐net hospital plan)	0.61	0.09 ± 1.11		−0.76	−1.30 ± −0.23	
% of Federal Poverty Level (FPL)						
0%–199%	2.69	1.69 ± 3.69	<0.001	1.55	0.92 ± 2.19	<0.001
200%–399%	0.77	−0.30 ± 1.80		1.03	0.43 ± 1.79	
400%–599%	0.94	−0.28 ± 2.10		0.76	0.04 ± 1.48	
600%–799%	0	−1.28 ± 1.28		−1.59	−2.64 ± −0.53	
800%–999%	0	−1.28 ± 1.28		−1.69	−2.76 ± −0.53	
≥1000% of FPL	Ref			Ref		
Cancer stage						
Stage I	Ref		0.006			
Stage II	0.07	−0.72 ± 0.86				
Stage III	0.74	−0.10 ± 1.58				
Stage IV	1.14	0.42 ± 1.86				

Abbreviations: BSLMC, Baylor St Luke's Medical Center; HHSC, Harris Health Smith Clinic.

## 4 DISCUSSION

This study developed and validated FinTox, a brief screening tool for identifying financial toxicity in cancer patients in clinical settings. Using a diverse sample from both private and safety‐net hospitals, the measure was constructed through qualitative interviews with patients and healthcare providers and enhanced by expert panel insights. FinTox, a 5‐item tool, measures patient financial strain, psychological impact, and care modifications. It distinguished financial toxicity across varied sociodemographic groups with high internal consistency, test–retest reliability, concept and concurrent validity. Additionally, evaluations comparing FinTox item endorsements to established COST thresholds for moderate to severe financial toxicity confirm its diagnostic accuracy and clinical utility.

Distinctions in content, focus, and response format likely influenced the magnitude of the correlation between FinTox and the COST. COST evaluates a wide range of financial concerns, from material conditions to concerns about future financial security. In contrast, FinTox assesses acute financial hardships, such as the immediate difficulties in affording care and meeting essential living expenses, thereby emphasizing its role in the prompt detection of financial distress within clinical settings. Moreover, the binary response format (yes/no) of FinTox, while facilitating clinical triage, could dampen the correlation with COST, which uses a Likert‐style response format that allows for more variable responses. These differences underscore that while both measures assess financial toxicity, they serve complementary roles. FinTox's streamlined design is optimized for rapid assessment and triage in clinical settings, while the COST, with its detailed scoring system and comprehensive assessment, may be better suited to the requirements of research environments.

Building on this, the diagnostic accuracy of FinTox, when compared against the more comprehensive COST thresholds, demonstrates its adequacy as a screening tool. It achieved a sensitivity of 72.3%, effectively identifying a large portion of individuals with moderate to severe financial toxicity. The specificity of 85.2% indicates that FinTox is effective in identifying 85.2% of individuals who do not have financial toxicity, potentially reducing the likelihood of unnecessary follow‐up assessments for those incorrectly flagged by less specific tools. The PPV of 83.2% suggests that a high proportion of those identified by FinTox truly experience financial distress (based on the COST), while the NPV of 70.3% indicates a moderate accuracy in identifying those free from such distress. These metrics collectively demonstrate that FinTox is an effective preliminary screening tool in clinical settings, accurately identifying individuals who may have financial toxicity and require further evaluation.

The study revealed notable disparities in FinTox scores when considering sociodemographic factors like race, income, and cancer stage. This aligns with existing research suggesting that financial toxicity disproportionately affects certain demographic groups.^41^, ^42^ Additionally, the varying levels of financial toxicity among patients from different hospital environments underscore the importance of targeted assessments and interventions to effectively address the financial burden in cancer care. The multivariable linear regression analysis identified key sociodemographic factors associated with patient financial toxicity. Race emerged as a significant determinant, with Black patients experiencing the highest levels of financial toxicity, likely due to systemic socioeconomic disparities and historical inequities in healthcare access exacerbating their financial vulnerability during cancer treatment.^43^ Conversely, Asian patients exhibited the lowest financial toxicity, having potentially benefited from cultural (such as family‐centric care models and multigenerational living arrangements) or socioeconomic factors.^44^ Income level also played a crucial role, with households earning below 600% of the FPL facing disproportionate financial burdens. For context, in 2023, the FPL for a family of four is $26,500; thus, 600% of this figure amounts to $159,000. This finding extends the understanding of financial toxicity beyond low‐income groups, indicating that middle and lower‐middle‐class families, earning too much for low‐income assistance but insufficient to cover high cancer treatment costs, are also significantly affected.^45^ This situation underscores the necessity for financial support and resources that cater to a broader socioeconomic spectrum.

The study revealed marked variations in financial toxicity based on healthcare settings and insurance types. Patients treated at HHSC, a safety‐net hospital, experienced greater financial toxicity compared to those at BSLMC, a private practice hospital. Notably, those with public health insurance reported less financial burden than individuals with private insurance. This implies that the lower care costs and reduced out‐of‐pocket expenses at safety‐net hospitals are inadequate in fully countering financial toxicity. These findings indicate that the economic impact of cancer care extends beyond direct medical expenses. Indirect costs, such as lost wages, contribute significantly to financial toxicity and often fall outside the direct purview of hospitals to intervene upon. This highlights the necessity for interventions beyond the healthcare system, emphasizing the role of community and employer‐based programs in addressing financial toxicity. It also suggests need for more community and employer based programs to address financial toxicity in addition to hospital based screening.

This study's strengths lie in its comprehensive approach, combining qualitative interviews with quantitative analysis, and involving an expert advisory group from diverse oncology‐related fields. The inclusion of patients from both private practice and safety‐net hospitals enriches the sample's representativeness, enhancing the FinTox measure's generalizability across various healthcare settings. The measure's solid psychometric properties and its effective initial validation further highlight its potential applicability in clinical settings. While the study's cross‐sectional nature limits the ability to establish causal relationships between financial toxicity and health outcomes, the aim of the study was to develop and validate the FinTox measure, not to assess its clinical applicability to assess outcomes. That said, future research should focus on longitudinal studies to assess the FinTox measure's sensitivity to financial toxicity changes over time. This would provide a more nuanced understanding of the financial challenges evolving throughout cancer care. Investigating and evaluating interventions designed to reduce financial toxicity, with effectiveness measured by FinTox scores, is also essential. Such research could significantly advance our understanding and management of financial toxicity in cancer care, offering critical insights for both clinicians and policymakers.

To enhance the integration of FinTox into clinical practice, developing clear guidelines and training for its use within existing clinical workflows is crucial. Pilot testing in real‐world settings will provide valuable insights into the practical challenges and benefits, guiding refinement efforts to ensure seamless integration without burdening healthcare providers. Additionally, given that any ‘yes’ response triggers further evaluation, focusing on refining the sensitivity and specificity of each item may enhance the measure's utility and efficiency in clinical settings. Such refinements could lead to more precise identification of patients who most need intervention, optimizing resource allocation and enhancing patient care.

In conclusion, FinTox marks a pivotal step in screening for financial toxicity in oncology care. Its streamlined and concise design is ideally suited for rapidly identifying patients experiencing financial hardship, thereby enabling prompt interventions that could positively impact treatment adherence and psychological well‐being. The measure effectively highlights disparities across patient demographics and healthcare settings, thus informing the creation of targeted strategies to alleviate financial toxicity. FinTox's reliability and practicality position it as an instrumental tool in improving patient outcomes, shaping healthcare policies, and enhancing HRQOL for cancer patients in diverse healthcare environments. Moreover, the insights garnered from this study hold the potential to influence policy changes aimed at reducing the financial burden of cancer care.

## AUTHOR CONTRIBUTIONS

**Hoda Badr:** Conceptualization (lead); formal analysis (lead); funding acquisition (lead); methodology (lead); project administration (lead). **Jessie Han:** Formal analysis (supporting). **Martha Mims:** Conceptualization (supporting); funding acquisition (supporting).

## CONFLICT OF INTEREST STATEMENT

None.

## PRECIS

The article presents the development and validation of FinTox, a novel tool designed for the efficient screening of financial toxicity in cancer patients within oncology clinical settings. Through qualitative interviews, expert panel consultations, and rigorous psychometric testing, the study demonstrates FinTox's reliability and validity.

## Supporting information


Appendix S1.

Appendix S2.

## Data Availability

The data that support the findings of this study are available from the corresponding author upon reasonable request.
