# Supplementary material for: Development and validation of FinTox: A new screening tool to assess cancer‐related financial toxicity
Source: Cancer Med. 2024 Aug 7;13(15):e7306. doi: 10.1002/cam4.7306 (PMC11306290; doi:10.1002/cam4.7306)
Supplement: Supplementary file 1 — Appendix S1. [file CAM4-13-e7306-s002.docx]

APPENDIX A - PATIENT INTERVIEW GUIDE

**FINANCIAL IMPACTS OF CANCER**

I would like to have a conversation with you about cancer and how it may have affected you and your family, financially.

- **First, tell me a little about your cancer. Probe: Type, stage, when diagnosed**
- **Tell me about what your general financial picture looked like at the time you were diagnosed with cancer.**
  - **What was your employment situation when you were diagnosed (Probe: full time, part time, self-employed, unemployed, retired)**
  - **Did you have health insurance coverage at the time that you were diagnosed?**
    - If yes, what type? (Probe: private, employer, self-paid). Were you satisfied with your insurance coverage of your cancer treatment? Why/why not? Do you still have insurance coverage? (if no, probe why)
  - **Since you were diagnosed, who handles the finances and paying any healthcare or medical bills? Has this changed since before cancer? How is this arrangement working out?**
- **Ask about food insecurity.** Some people that we have talked to reported having trouble buying healthy food or not having enough food to eat while they were being treated for cancer. Is this something that you ever experienced?”
  - If No, probe: Was food something that you prioritized in your budget while you were being treated for cancer?”
  - If yes, Probe: what strategies did you use when food supplies were insufficient and money was not available to acquire food?
- **Ask about housing insecurity.** Some people that we have talked to reported having trouble paying their rent or mortgage payments while they were being treated for cancer. Is this something that you ever experienced?”
  - If No, probe: Was housing something that you prioritized in your budget while you were being treated for cancer?”
  - If yes, Probe: what strategies did you use when you were worried about making rent/housing payments while you were undergoing treatment for cancer?
- **For people who are/were employed at time of diagnosis:**
  - How has cancer affected your work experience? PROBE: did you take off time from work, coming in late, leaving early, cut back on hours, get passed up for a promotion
  - How did you balance cancer treatment and work responsibilities?
  - Does your employer know about your cancer? What has their reaction been like?
  - Has your employer offered any work flexibility? (PROBE: Flexible work hours; Paid sick days; Paid leave,; Programs like information, referrals, counseling, or an employee assistance program, to help caregivers like yourself; Telecommuting or working from home). What has been most helpful?
  - Since your diagnosis, have you made any changes in your employment? PROBE: resigned, went part time, changed jobs? Was this your decision or did the change come from your employer? What prompted the change?
- **Since you were diagnosed with cancer, have you encountered any out-of-pocket expenses that you did not expect or things you had to pay for yourself?** If so, what were they? PROBE: chemotherapy drugs, prescription medications? Parking, childcare. How did you handle that situation?
- **What, if any, methods have you used to offset costs of care and expenses? PROBES:**
  - - use personal savings?
    - take loans, use credit cards, or second mortgages?
    - borrow money from friends or family?
    - cut back on vacations/leisure activities
    - cut back on budget for food/clothing?
- **Did the cost of your medical care affect medical decisions in any way? PROBES:**
  - Did cost play a role in your/your loved one‘s decisions about cancer treatment? Tell me about that.
  - Did you ever not receive, limit, or stop treatment because of concerns about your ability to pay for it? (Probe: Tell me more about that.)
  - Did you ever not fill a prescription due to cost or take less than the recommended dose of medcine so that it would last longer? Tell me more about that.
  - How about indirect costs of care like transportation costs, childcare costs, etc. affected your ability to attend medical appointments or receive treatment? Can you tell me about that? How do you manage?
- **How has dealing with the financial aspects of your medical care affected you and your family? PROBES:**
  - When did you first start to experience financial hardship related to your cancer? (Probe: Can you tell me more about that?)
  - When did you reach your highest point of financial hardship, and what was that like for you?
  - Did you talk to anyone about the financial impacts of cancer? e.g., doctor, private support, professional support? Was it helpful?
- **Looking back on your finances and overall financial quality of life before your cancer diagnosis, what has changed? What are your greatest financial concerns right now?**
- **What advice would you give to the healthcare system (hospitals, cancer centers, insurance companies, government) to help patients and caregivers dealing with financial aspects of cancer? What intiatives would be helpful?**
