# Supplementary material for: Development and validation of FinTox: A new screening tool to assess cancer‐related financial toxicity
Source: Cancer Med. 2024 Aug 7;13(15):e7306. doi: 10.1002/cam4.7306 (PMC11306290; doi:10.1002/cam4.7306)
Supplement: Supplementary file 2 — Appendix S2. [file CAM4-13-e7306-s001.docx]

**Supplementary Appendix B_Provider Interviews**

1. Can you tell me a little bit about the need for financial navigation services for your patients?
   1. What are some of the most common issues or concerns that patients bring to you?
      1. What are some barriers for accessing financial navigation services?
   2. How do you think financial concerns impact their adherence to cancer treatments?
      1. Quality of life?
      2. What are the greatest needs that you see?
   3. As a healthcare provider, what do you see as your role in addressing this?
2. Can you tell me a little bit about how patient financial concerns are addressed in your clinic?
   1. What services are currently being offered?
   2. Are you satisfied with the type of services being offered?
   3. What could be improved?
   4. What is the biggest barrier to improving financial navigation?
3. Who should have a discussion with the patient about financial concerns?
4. Who should have cost of care conversations with patients?
5. When should cost of care conversations occur?
   1. When would it be appropriate or not appropriate to have these conversations?
6. How often should the cost of care conversations happen?
   1. What should or should not be discussed?
   2. How should these discussions unfold and information be delivered?
7. We are in the process of developing a screening measure for financial distress to systematically identify patients who are in need of financial navigation services.
   1. Do you think a tool like this would be helpful?
      1. Why or why not
   2. What kind of infrastructure would be needed to successfully implement something like this?
   3. What is standing in the way of implementation?
   4. How can we overcome these barriers?
   5. Who are the key players who would need to buy in to improving financial navigation services?
   6. What metrics are important?
      1. (example: # patients referred to financial navigation)
   7. If you had a magic wand: how would you improve how we measure financial concerns?
   8. Who else should we talk to?
   9. Is there anything else we didn’t ask?
